# Glycerophosphodiester Phosphodiesterase Identified as Non-Reliable Serological Marker for *Borrelia miyamotoi* Disease

**DOI:** 10.3390/microorganisms8121846

**Published:** 2020-11-24

**Authors:** Michael Reiter, Theresa Stelzer, Anna M. Schötta, Mateusz Markowicz, Michael Leschnik, Anna Harsch, Edda Reiß, Richard E. Kneusel, Hannes Stockinger, Gerold Stanek

**Affiliations:** 1 Institute for Hygiene and Applied Immunology, Center for Pathophysiology, Infectiology and Immunology, Medical University of Vienna, 1090 Vienna, Austria; theresa.stelzer@meduniwien.ac.at (T.S.); anna-margarita.schoetta@meduniwien.ac.at (A.M.S.); mateusz.markowicz@meduniwien.ac.at (M.M.); hannes.stockinger@meduniwien.ac.at (H.S.); gerold.stanek@meduniwien.ac.at (G.S.); 2 University Clinic for Small Animals, Clinical Unit of Internal Medicine Small Animals, University of Veterinary Medicine, 1210 Vienna, Austria; michael.leschnik@vetmeduni.ac.at; 3 DIARECT AG, 79111 Freiburg, Germany; anna.harsch@diarect.com (A.H.); edda.reiss@diarect.com (E.R.); richard.kneusel@diarect.com (R.E.K.)

**Keywords:** borrelia, tick-borne, miyamotoi, lyme, relapsing fever, seroprevalence

## Abstract

The relapsing fever group *Borrelia miyamotoi* is an emerging tick-borne pathogen. Diagnosis of infection is currently mainly based on serological methods detecting antibodies against *B. miyamotoi* glycerophosphodiester phosphodiesterase (GlpQ). Here, we scrutinized the reliability of GlpQ as a diagnostic marker and compared the seroprevalence in different study populations and by applying various immunoblotting methods. Antibodies were detected in the sera of 7/53 hunters and in 1/11 sera of Lyme neuroborreliosis patients. Furthermore, 17/74 sera of persons with high concentrations of anti-Borrelia burgdorferi sensu lato (α-Bbsl) antibodies reacted strongly with *B. miyamotoi* GlpQ in immunoblots. The *B. miyamotoi* GlpQ seroprevalence was 7/50 in α-Bbsl negative persons. In healthy blood donors from commercial suppliers and from the Austrian Red Cross, seroprevalences were 5/14 and 10/35, respectively. Strikingly, two *B. miyamotoi* PCR-positive cases from Austria had negative GlpQ serology, indicating poor sensitivity. Finally, when we analyzed sera of dogs, we found α-*B. miyamotoi* GlpQ antibody seroprevalence in tick-free dogs (*n* = 10) and in tick-exposed dogs (*n* = 19) with 2/10 and 8/19, respectively. Thus, our results indicate that GlpQ-based *B. miyamotoi* serology holds neither specificity nor sensitivity.

## 1. Introduction

In 2011, reports from Russia and case reports from the United States, Europe and Japan associated human febrile disease with infection by *B. miyamotoi* [1,2,3,4,5,6,7,8,9]. The clinical presentations were often unspecific with symptoms such as fever, fatigue, headache, and chills [10]. Meningoencephalitis due to *B. miyamotoi* was reported in severely immuno-compromised patients [6,8]. However, an agreed case definition has not yet been established for *B. miyamotoi* disease. Interestingly, *B. miyamotoi*, a relapsing fever (RF) group *Borrelia*, is vectored by hard ticks of the *Ixodes ricinus* complex, which is also the vector of *B. burgdorferi* sensu lato (Bbsl) spirochetes, to which the agents of Lyme borreliosis belong [11]. This unusual host for an RF *Borrelia* coined the term hard tick-borne relapsing fever (HTBRF) for this spirochetal disease [2]. This might have led also to confusion with the agent of Lyme borreliosis in the early years and the delay of recognition of *B. miyamotoi* as a human pathogen [5].

The gene for glycerophosphodiester phosphodiesterase (*glpQ*), encoding GlpQ, an enzyme employed in the phospholipid synthesis, is present in RF borreliae but not in Lyme borreliae [12,13]. Both the gene and its gene product have been used in the recent past to detect *B. miyamotoi*. Direct detection from samples such as blood and cerebrospinal fluid by PCR targeting the glpQ gene has been achieved in a number of patients, who presumably suffered from HTBRF [1,8,9,14]. The GlpQ protein has been used in enzyme immunoassays for the detection of specific antibodies against *B. miyamotoi* in patients as well as in seroprevalence studies [3,4,10,15]. Additionally, variable major proteins (VMPs) have been suggested as antigens in the serological detection of *B. miyamotoi* [16]. Seroprevalence of *B. miyamotoi* has been assessed in the Netherlands, where it was found in 2% of blood donors, whereas it was significantly higher in a population of forestry workers (10%) and patients with suspected human granulocytic anaplasmosis (14.6%) [15]. However, the presence of specific antibodies against *B. miyamotoi* constitutes an indirect proof of infection that does not necessarily point to an active infection.

We previously reported the presence of *B. miyamotoi* in 1% of Austrian *I. ricinus* ticks [17]. Consequently, in the present study we assessed the seroprevalence of this spirochete in populations with different exposure to ticks as well as in two persons with PCR proven *B. miyamotoi* infection. Additionally, we had access to sera of dogs that had been raised in a tick-free environment, as well as to sera of dogs regularly exposed to ticks. 

## 2. Study Participants, Material and Methods

### 2.1. Human Sera

Serum specimens were obtained from 53 hunters, 11 patients with confirmed Lyme neuroborreliosis (LNB), and 74 persons with high concentration of α-Bbsl IgG antibody (>100 AU/mL; Medac, Wedel, Germany). Serum specimens from 50 persons without IgG antibodies (<12.6 AU/mL; Medac, Germany) to Bbsl were used as controls. Furthermore, serum samples of 35 blood donors from Eastern Austria (the federal states of Lower Austria, Vienna, and Burgenland) obtained from the Austrian Red Cross were included, as well as 14 additional serum samples of healthy blood donors obtained from commercial serum suppliers. We also tested sera taken at two time points from a patient with proven *B. miyamotoi* infection. Finally, we tested sera from a person bitten by a tick, who also tested positive for *B. miyamotoi* by PCR of blood. From this person we tested serum taken at the time point of positive PCR and serum taken two months before and three months after the tick bite, respectively.

### 2.2. Dog Sera

We collected 20 serum samples from 10 Beagles raised under tick-free conditions, and 19 sera from tick-exposed dogs (mixed breeds). The ten non-exposed dogs had originally participated in a vaccination study with a Lyme borreliosis vaccine and blood samples taken before and after vaccination were used.

### 2.3. Bacterial Strains and Growth Conditions

Bacterial strains and sources are given in Table 1. *Escherichia coli* strains were cultivated in lysogeny broth medium (per liter: 10 g peptone, BD, Austria; 5 g NaCl; 5 g yeast extract, Sigma-Aldrich, Austria) at 37 °C. *Borrelia afzelii* and *B. turicatae* were grown in a modified BSK II medium, as previously described [17]. Upon receipt, *B. miyamotoi* strains were grown as previously described in a modified Kelly–Pettenkofer medium containing fetal calf serum [18]. However, these strains grew only for a few passages in our laboratory, probably due to incompatibility to the fetal calf serum used. Nevertheless, initial cultures yielded enough cells for preparing whole-cell lysates in order to continue the study.

### 2.4. Recombinant Expression and Purification of B. miyamotoi Proteins

The sequence of the *B. miyamotoi* CT14D4 gene coding for GlpQ (GenBank: CP010308.1, 248,426–249,430) was synthesized without its N-terminal signal peptide as previously reported by others [19]. Subsequently it was cloned into expression vector pET28b(+) (Merck, Vienna, Austria) for production of GlpQ carrying a C-terminal 6x histidine (His-) tag (GlpQ-C). We also constructed an N-terminally tagged version of GlpQ (GlpQ-N), as we were concerned that the tags together with the linker peptide that comes from the vector might interfere with the recognition by IgG antibodies. To this end, we cloned the respective sequence into the pRSET-B vector (Fisher Scientific, Austria) using standard cloning methods. We also produced a recombinant version of the *B. miyamotoi* variable small protein 1 (Vsp1). This protein belongs to the VMP family of *Borrelia* proteins and has recently been suggested as an additional marker for detection of α-*B. miyamotoi* antibodies [16]. For expression of Vsp1, the respective gene was PCR amplified from *B. miyamotoi* strain LB-2001 and cloned into expression vector pRSET-B to add the His-tag at the N-terminus (Fisher Scientific, Vienna, Austria). 

All clones were verified by sequencing (sequence information is given in the supplemental material). Sequencing of the Vsp1 construct confirmed the same point mutations in comparison to the published sequence (GenBank KF031441) that have been reported earlier [16]. The respective expression vectors were transformed into *E. coli* expression strain Bl21(DE3)pLysS. The recombinant proteins were then expressed and purified. The quality of purification was evaluated by SDS-polyacrylamide gel electrophoresis (SDS-PAGE) followed by staining with Coomassie Brillant Blue (Figure S1). Additionally, a commercially available version of recombinant GlpQ (GlpQ_DIA_) produced by DIARECT AG (Freiburg, Germany) was used. This version uses the coding sequence of *B. miyamotoi* strain FR64B and is produced in *E. coli* with an N-terminal 10x His-tag (sequence given in the supplemental information). 

### 2.5. SDS-PAGE and Western Blotting

Purified proteins or whole cells were mixed with Laemmli sampling buffer, separated by SDS-PAGE and blotted onto a nitrocellulose membrane. After blocking with skim milk powder in Tris-buffered saline buffer containing 0.05% Tween 20 and washing, the membranes were incubated with human or dog sera and subsequently with the respective secondary antibodies (either goat-anti-human-IgG-Fc-horseradish peroxidase (HRP)-conjugate or a goat-anti-dog-IgG-HRP-conjugate).

### 2.6. Membrane-Based Macroarray and Western Blot Strip Analysis

Recombinant GlpQ (DIARECT) was diluted to 0.1 mg/mL, 0.2 mg/mL and 0.4 mg/mL in phosphate buffer. Spots of approximately 12 nL were printed onto a nitrocellulose membrane glued onto a microscope slide. Positive control spots (human IgG and goat-anti-human IgG+IgM+IgA (H+L), and negative control spots (human serum albumin, HSA), as well as buffer control spots, were also included. After blocking and washing, the arrays were incubated with the serum samples and subsequently either with goat-anti-human-IgG-Fc-alkaline-phosphatase—(AP)-conjugate or a rabbit-anti-dog-IgG-Fc-AP-conjugate. For further analysis of a subset of the tested sera, Western blot strips were prepared. To this end, recombinant GlpQ (DIARECT) was separated by standard SDS-PAGE and blotted onto a 0.2 µm nitrocellulose membrane. After blocking with skim milk and washing, the membrane was dried and cut into strips. These were incubated with the sample sera and respective secondary antibodies as before. 

### 2.7. Detection of B. miyamotoi DNA 

Detection of *B. miyamotoi* DNA was conducted as previously described [20]. Briefly, DNA was extracted from EDTA blood followed by qPCR targeting the *B. miyamotoi glpQ* gene. For further confirmation, PCRs targeting the 16–23S intergenic spacer region, the 16S rRNA coding region and the *glpQ* gene were performed and the amplicons were sequenced (Microsynth, Wolfurt, Austria).

### 2.8. Ethical Approval

The patient with the PCR proven *B. miyamotoi* infection [20] signed a declaration of consent. The PCR positive person with a tick bite participated in a study approved by the institutional ethics committee (Medical University of Vienna, Vienna, Austria; study number 1064/2015).

All other human samples included in this study were retrieved from the archives of diagnostic institutions. Samples were fully anonymized. The sole information linked to the samples were the results of commercially available Lyme borreliosis diagnostic assays and the result of the immunoblots developed in the present study.

The animal experiments were approved by the institutional ethics committee (University of Veterinary Medicine, Vienna, Austria) and the Austrian Ministry for Science and Research (GZ68.205/0148-C/GT/2007 and GZ68.205/25-II/10b/2010).

## 3. Results

**Figure 1:**
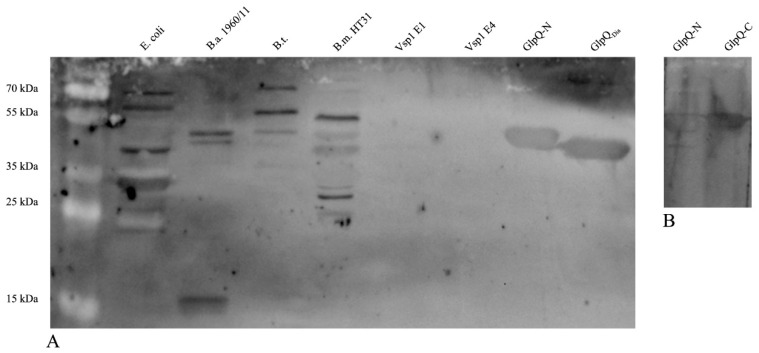
(**A**) Representative immunoblot with serum of a hunter. The sample was probed for the presence of IgG antibodies against lysates of the *E. coli* expression strain Bl21(DE3)pLysS and different *Borrelia* species as well as purified recombinant proteins Vsp1 and GlpQ. *E. coli* strain Bl21(DE3)pLysS; B.a., *B. afzelii* strain 1960/11; B.t., *B. turicatae*; B.m. HT31, *B. miyamotoi* strain HT31; Vsp1 of different purities (E1 and E4); GlpQ-N, N-terminally 6xHis-tagged version of GlpQ produced in the Vienna laboratory; GlpQ_DIA_, recombinant GlpQ produced by DIARECT. (**B**) Immunoblot of the same serum sample to test whether the position of the His-tag (GlpQ-N, N-terminal 6x His-tag; GlpQ-C, C-terminal 6x His-tag) has influence on reactivity.

For detection of *α-B. miyamotoi* antibodies, we used one Vsp1- and three differently produced GlpQ preparations. Recombinant GlpQ produced by DIARECT contained an N-terminal 10x His-tag. The Vienna laboratory produced in addition to an N-terminal 6xHis-variant also one with a C-terminal 6xHis-tag to analyze whether there is a difference in the reactivity when the tag is swapped (Figure 1B). Vsp1 was also produced in the Vienna laboratory. The samples were run over SDS-PAGE and after transfer to the membranes, the reactive antibodies were visualized by immunostaining.

As initial test groups, we selected persons with a high risk of exposure to ticks. We screened sera of hunters from the federal state of Burgenland, Austria, for the presence of IgG antibodies to *B. miyamotoi* GlpQ-N/-C. Seven out of the 53 samples were positive (Table 2). Two of the seven samples were additionally tested in a more detailed immunoblot containing also crude lysates of the following bacteria: (1) *E. coli* expression strain Bl21(DE3)pLysS, (2) *B. afzelii* strain 1960/11, (3) *B. turicatae*, (4) *B. miyamotoi* strain HT31, and (5) recombinant *B. miyamotoi* Vsp1 protein. We found the strongest reactions with *E. coli* total lysate, and milder ones with crude lysates of *B. miyamotoi*, *B. turicatae*, and *B. afzelii*. We did not observe any reactivity towards recombinant Vsp1. A representative immunoblot obtained with one of the two hunters is shown in Figure 1A.

### 3.1. α-B. miyamotoi Antibodies in Humans 

Next, we tested sera of patients with confirmed LNB. One patient scored positively, and this serum was also tested in a more detailed immunoblot (Figure S2). In addition, we tested sera of patients who had high concentrations of α-Bbsl IgG. As control groups, we selected serum samples of persons who were negative for α-Bbsl IgGs, and sera of healthy blood donors from two different sources: (a) from the Austrian Red Cross and (b) from commercial suppliers. The Austrian samples were analyzed in Vienna; the samples from the commercial supplier by DIARECT in Freiburg. The results are shown in Table 2.

### 3.2. α-B. miyamotoi Antibodies in Dog Sera 

As the next cohort of serum donors, we tested dogs kept under normal but also under tick-free conditions. When using GlpQ-N, eight out of 19 dogs who had regular exposure to ticks showed a positive reaction. These dogs participated in a natural exposure/infestation study and all dogs were proven to be infested by up to 75 ticks within one year prior to blood sampling. We had also the opportunity to test ten dogs raised under tick free conditions. They originally participated in a Lyme vaccination study and serum had been sampled before and after vaccination. Before vaccination, one dog tested weakly positive for GlpQ. After vaccination, another dog tested positive. Hence, the serum of two out of these ten dogs scored positively with GlpQ. However, each of these sera was only positive at one of the two available time points (Table 2).

### 3.3. Confirmation of Results by Blinded Testing

**Figure 2:**
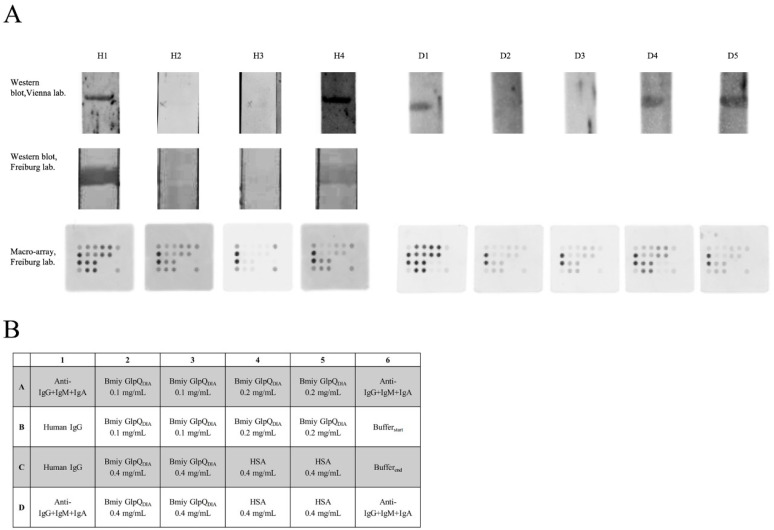
(**A**) Representative results of blind testing of human (H1–4) and dog sera (D1–5). First row. Immunoblot results from the Vienna laboratory. Second row. Immunoblot results from the DIARECT laboratory. Third row. Results from the membrane-based macroarray. (**B**) Spot layout of the macroarray developed by DIARECT. Different concentrations of *B. miyamotoi* GlpQ_DIA_ (Bmiy_GlpQ_DIA_) were applied to the array. Human IgG and goat-anti-human IgG+IgM+IgA served as positive controls. Human serum albumin (HSA) and buffer controls at the start and end of the spotting procedure are also included.

For inter-lab confirmation of the test results, we selected four human samples (two positive—H1 and H4, two negative ones—H2 and H3) and five dog sera (three positive–D1, D4, D5, two negative—D2, D3) that were tested blindly by DIARECT. Additionally, a subset of the sera was tested via Western blot strip analysis (Figure 2A,B). The human sample H2, negative in the Vienna lab, scored positively in the DIARECT macroarray (Figure 2A). However, the same sample was negative when DIARECT tested it in the Western blot strip analysis, thereby mirroring the results of Vienna. Regarding the dog sera, 4 out of 5 sera gave identical results in both labs (D1–D4), while serum D5 tested positive in the Vienna lab, but negative in the DIARECT microarray (Figure 2A).

### 3.4. Serum Analysis of Persons with PCR Confirmed B. miyamotoi Infection

**Figure 3:**
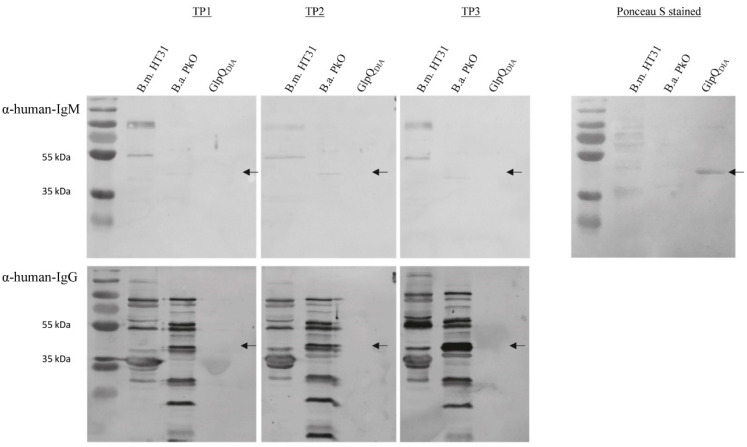
Immunoblot with the serum of the patient with PCR-confirmed *B. miyamotoi* infection using crude lysate of *B. miyamotoi* strain HT31 (B.m. HT31), *B. afzelii* strain PkO (B.a. PkO) and recombinant GlpQ produced by DIARECT (GlpQ_DIA_). Three time points (TP) are shown. At TP2 the blood sample tested positively for *B. miyamotoi* by PCR; TP1 was two months before and TP3 three months afterwards. The upper panel was probed with α-human-IgM antibodies, the lower one with α-human-IgG antibodies. Arrows indicate the correct height of GlpQ. Right panel. Representative Ponceau S stain of one of the membranes.

We analyzed sera collected from a person, whose blood yielded a *B. miyamotoi* positive PCR after the bite of a tick. From this person, serum collected two months before and three months after the tick bite was also tested. The person did not report any symptoms at the time point of positive PCR, nor at the follow-up and did not receive medical treatment. Strikingly, none of these samples showed a reaction to GlpQ_DIA_, although multiple reactions with *B. miyamotoi* and *B. afzelii* whole-cell lysates were visible (Figure 3).

Finally, we analyzed the serum of the first patient confirmed by PCR to be infected with *B. miyamotoi* in Austria [20]. From this patient we analyzed serum samples taken at two time points. One was collected at the time point of PCR positivity. The second had been taken 13 weeks earlier for other reasons and the sample was sent to us for routine testing. Using GlpQ_DIA_, the immunoblots of the earlier time point displayed faint IgM and IgG signals; at time point two, the IgM signal had disappeared completely, and the IgG signal was barely visible. Multiple reactions were visible in whole-cell lysates of *B. miyamotoi* isolates LB-2001 and HT31 and *B. afzelii* (Figure S3). 

## 4. Discussion

Upon screening sera of hunters for reactivity with GlpQ, 13.2% scored positively; the outcome thus being concordant with a previous study in the Netherlands [15]. In addition, the percentage of positive sera of the LNB patients was comparable to that of the hunter population and was in agreement with the Dutch study [15]. Although the LNB population is too small to draw a definitive conclusion, it adds together with the hunter cohort to the assumption that co-infections with Bbsl and *B. miyamotoi* may occur [7]. The results with the sera of patients, who had high antibody titers against Bbsl, further support co-infection at first glance. Sequential infection with both species is also possible, given the fact that *α-B. burgdorferi* antibodies may persist for years [21,22]. However, the Bbsl seronegative control groups displayed a similarly high rate of GlpQ-positive immunoblots. When analyzing the Bbsl seropositive versus seronegative groups statistically, we found no significant difference. We found this substantial seroprevalence of α-*B. miyamotoi* GlpQ IgG in our general population by independent testing using different methods in the Vienna- and Freiburg laboratories. This result is in stark contrast to the study authored by Jahfari et al., in which only 2% of blood donors tested positively [15]. This raises the question whether the prevalence of *B. miyamotoi* antibodies varies in different geographic areas or whether there is simply considerable cross-reactivity with GlpQs from other bacterial species (e.g., *E. coli*, *B. subtilis*) including other spirochetes. The infection rate of Ixodes ticks with *B. miyamotoi* in Austria, as in other European countries, is approximately 1% [17,23,24,25]. The seroprevalences of *B. miyamotoi* might be higher than expected from this infection rate of ticks, because in contrast to Bbsl, *B. miyamotoi* is transovarially transmitted from female carriers. Thus, all tick stages are relevant for infection in humans [26]. Furthermore, persistence of α-*B. miyamotoi* GlpQ IgG after infection, as sometimes observed with antibodies against Lyme borreliae [22], might also occur. However, we are of the opinion that seroprevalences as high as in our present study cannot be explained by the latter mechanisms but rather by cross-reactivity. As RF borreliae GlpQs share only approximately 30–50% similarity with homologous genes of other bacteria [12], it is commonly thought that cross-reactivity to GlpQ homologs of other pathogens is insignificant [5]. However, cross-reactivities among GlpQs of RF group borreliae have been predicted and reported [12,19] and may include *B. miyamotoi* as well. Furthermore, our finding that dogs bred under tick-free conditions were also positive, strongly points to the frequently false-positive detection of α-GlpQ-IgG and supports the cross-reactivity hypothesis. In the tick-free group, two time points (before and after vaccination against Lyme borreliosis) were tested for each dog. For two dogs, only one single time point was positive, thus arguing for a rather unspecific result that might have arisen from cross-reactions, presumably with other spirochetes. This could also explain the considerably higher number of positive sera in the tick-exposed group than in the tick-free group as a cross-reaction with GlpQs of other tick-transmitted bacteria. Theoretically, transmission of *B. miyamotoi* to the dogs could have been achieved by vectors other than ticks. However, currently there is no evidence for this. 

We initially tested the sera also for the presence of IgG antibodies against Vsp1, another *B. miyamotoi* antigen (Figure 1). However, as we saw no reaction with this antigen, we omitted Vsp1 for the sake of faster screening. We consider that the Vsp1 result reflects the infection rate with *B. miyamotoi* in our regions. As an argument against this hypothesis, one could use the high variability of Vsp1 and speculate that it induces specific antibodies, which decline after neutralizing and clearing the respective serotype from the blood of the infected host [16,27]. Further, we used recombinant Vsp1 from strain LB-2001, which was isolated in North America and could express a Vsp1 variant not recognized by our sample group.

## 5. Conclusions

In summary, we found a considerable seroprevalence of GlpQ IgG not only in tick-exposed populations but also in the control groups and even in dogs bred tick-free. Importantly, we only detected extremely faint signals for α-GlpQ antibodies in an HTBRF-diagnosed Austrian patient with a *B. miyamotoi*-positive PCR. In another PCR-positive person, we saw no specific reaction, including a sample taken three months after PCR confirmation. This further diminishes the diagnostic value of GlpQ serology, although certainly more similar cases would have to be studied to draw a definitive conclusion. Taken together, our results show that care should be taken in diagnosis of HTBRF using GlpQ as a single marker. Additional antigens, e.g., the VMPs of *B. miyamotoi*, should be evaluated as diagnostic markers to ensure sufficient specificity for reliable diagnosis.

## Figures and Tables

**Table 1:** Bacteria used in this study.

Bacterial Strain	Source
*E. coli* Top10F’	Thermo Fisher Scientific, Vienna, Austria
*E. coli* BL21(DE3)pLysS	Thermo Fisher Scientific, Austria
*B. miyamotoi* HT31	Courtesy of J. Hovius, Center for Experimental and Molecular Medicine, Academic Medical Center, Amsterdam, the Netherlands
*B. miyamotoi* LB-2001	Courtesy of J. Hovius, Center for Experimental and Molecular Medicine, Academic Medical Center, Amsterdam, the Netherlands
*B. afzelii* 1960/11	Courtesy of E. Ruzic-Sabljic, Institute of Microbiology and Immunology, Faculty of Medicine, University of Ljubljana, Ljubljana, Slovenia
*B. turicatae*	Courtesy of V. Fingerle, National Reference Center for Borrelia, Oberschleißheim, Germany

**Table 2:** Results of serum screenings.

Test Group Sera from:	Number of Positives	Relative Positivity
Hunters from federal state Burgenland	7/53	13.2%
Neuroborreliosis patients	1/11	9.1%
Donors with high titers of α-Bbsl antibodies (>100 AU/mL)	17/74	23%
Donors negative for α-Bbsl antibodies ^1^ (<12 AU/mL)	7/50	14%
Healthy blood donors from Austria screened in Vienna	10/35	28.6%
Healthy blood donors screened in Freiburg	5/14	35.7%
Non-tick exposed dogs before vaccination	1/10	10%
Non-tick exposed dogs after vaccination	1/10	10%
Tick exposed dogs	8/19	42.1%

^1^ Samples negative in EIA testing.

## Supplementary Materials

The following are available online at https://www.mdpi.com/2076-2607/8/12/1846/s1. Figure S1: SDS-polyacrylamide gel electrophoresis of the different versions of GlpQ produced by us followed by staining with Coomassie Brilliant Blue. Figure S2: Immunoblot with the serum of the LNB patient, whose IgG shows weak reactivity against GlpQ. Figure S3: Serum samples of a *B. miyamotoi*-infected person from two different time points (TP).
