# Supplementary material for: Glycerophosphodiester Phosphodiesterase Identified as Non-Reliable Serological Marker for Borrelia miyamotoi Disease
Source: Microorganisms. 2020 Nov 24;8(12):1846. doi: 10.3390/microorganisms8121846 (PMC7760085; doi:10.3390/microorganisms8121846)
Supplement: Supplementary file 1 [file microorganisms-08-01846-s001.pdf]

Supplemental information

Sequences of constructs

Nucleotide and amino acid sequences of GlpQ constructs from *B.* miyamotoi are given below. Expression vectors pET28b(+), pRSET-B, or pET24d were used. The resulting recombinant proteins carry either a C- or an N-terminal 6xHis-tag or, in the case of pET24d, an N-terminal 10xHis-tag. Sites for restriction endonucleases are underscored. The starting codon (methionine) and the His-tag are marked by bold letters.

pET28b(+)_Bm_GlpQ-C

1 CC**ATG**GCCTT TAAACAAGAA ATGGGTGAAA ACAAAAAGTC ACCATTGATC ATAGCTCACA
61 GGGGTGCTAG TGGATACCTT CCAGAACATA CCTTAGAAGC TAAAGCATAT GCTTATGCCT
121 TAGGAGCTGA TTATCTAGAA CAAGACATAG TTCTAACAAA GGACAATATT CCTGTTATAA
181 TGCACGACCC AGAAATTGAC ACAACCACAA ATGTTGCACA ATTATTTCCC AATCGAGCTA
241 GAGAAAACGG AAGATATTAC GCTACTGACT TCACACTAAC TGAACTTAAA TCACTAAATC
301 TCAGTGAAAG ATTTGATCCT GAAAATAAAA AACCAATATA TCCTAATCGT TTTCCCTTAA
361 ATGAATATAA TTTTAAAATT CCAACTTTAG AAGAAGAAAT ACAATTCATA CAAGGACTAA
421 ATAAAAGCAC AGGAAAGAAT GTTGGGATTT ACCCTGAGAT TAAAAAACCC TTCTGGCATA
481 AACAACAAGG TAAAGATATC TCTAAAATTG TAATAGAAAT TTTAAATAAA TATGGGTATA
541 AATCAAAAGA AGATAAGATT TACCTACAAA CATTCGACTT TGATGAATTA AAAAGAATAA
601 GAAAAGAACT TGGATACCAA GGCAAATTAA TAATGCTTGT TGGAGAAAAT GACTGGAATG
661 AAGCACCAAC AGACTATGAA TATATAAAAT CAGAAGAAGG TATTGCTGAA GTTGCAAAAT
721 ATTCTGATGG AATTGGACCC TGGATACCCC AAATTATAAT TGATGGTAAA ATAACAGAAC
781 TTACAAACTT AGCACATAAA TATAATATAG AGGTTCATCC TTATACATTT AGGACAGATG
841 CATTACCTTC ATATGTTAAA AATGAAAATG AATTATTAGA TTTATTATTT AACAAAGCAA
901 AAGTAGATGG TATATTTACA GATTTTACTG ACACAGTAAT GAATTTCATA AAAAAAGAAT
961 TCCTTGAAGT TCTTTTTCAA GGTCCT**CACC ACCACCACCA CCAC**GAATTC TAAAAGCTT

1 **M**AFKQEMGEN KKSPLIIAHR GASGYLPEHT LEAKAYAYAL GADYLEQDIV LTKDNIPVIM
61 HDPEIDTTTN VAQLFPNRAR ENGRYYATDF TLTELKSLNL SERFDPENKK PIYPNRFPLN
121 EYNFKIPTLE EEIQFIQGLN KSTGKNVGIY PEIKKPFWHK QQGKDISKIV IEILNKYGYK
181 SKEDKIYLQT FDFDELKRIR KELGYQGKLI MLVGENDWNE APTDYEYIKS EEGIAEVAKY
241 SDGIGPWIPQ IIIDGKITEL TNLAHKYNIE VHPYTFRTDA LPSYVKNENE LLDLLFNKAK
301 VDGIFTDFTD TVMNFIKKEF LEVLFQGP**HH HHHH**EF

pRSET-B_Bm_GlpQ-N

1 **ATG**CGGGGTT CT**CATCATCA TCATCATCAT** GGTATGGCTA GCATGACTGG TGGACAGCAA
61 ATGGGTCGGG ATCTGTACGA CGATGACGAT AAGGATCCGA GCTCGAGATC TGCAGCTGGT
121 ACCATGGCCT TTAAACAAGA AATGGGTGAA AACAAAAAGT CACCATTGAT CATAGCTCAC
181 AGGGGTGCTA GTGGATACCT TCCAGAACAT ACCTTAGAAG CTAAAGCATA TGCTTATGCC
241 TTAGGAGCTG ATTATCTAGA ACAAGACATA GTTCTAACAA AGGACAATAT TCCTGTTATA
301 ATGCACGACC CAGAAATTGA CACAACCACA AATGTTGCAC AATTATTTCC CAATCGAGCT
361 AGAGAAAACG GAAGATATTA CGCTACTGAC TTCACACTAA CTGAACTTAA ATCACTAAAT
421 CTCAGTGAAA GATTTGATCC TGAAAATAAA AAACCAATAT ATCCTAATCG TTTTCCCTTA
481 AATGAATATA ATTTTAAAAT TCCAACTTTA GAAGAAGAAA TACAATTCAT ACAAGGACTA
541 AATAAAAGCA CAGGAAAGAA TGTTGGGATT TACCCTGAGA TTAAAAAACC CTTCTGGCAT
601 AAACAACAAG GTAAAGATAT CTCTAAAATT GTAATAGAAA TTTTAAATAA ATATGGGTAT
661 AAATCAAAAG AAGATAAGAT TTACCTACAA ACATTCGACT TTGATGAATT AAAAAGAATA
721 AGAAAAGAAC TTGGATACCA AGGCAAATTA ATAATGCTTG TTGGAGAAAA TGACTGGAAT
781 GAAGCACCAA CAGACTATGA ATATATAAAA TCAGAAGAAG GTATTGCTGA AGTTGCAAAA
841 TATTCTGATG GAATTGGACC CTGGATACCC CAAATTATAA TTGATGGTAA AATAACAGAA
901 CTTACAAACT TAGCACATAA ATATAATATA GAGGTTCATC CTTATACATT TAGGACAGAT
961 GCATTACCTT CATATGTTAA AAATGAAAAT GAATTATTAG ATTTATTATT TAACAAAGCA

1021 AAAGTAGATG GTATATTTAC AGATTTTACT GACACAGTAA TGAATTTCAT AAAAAAAGAA
1081 TTCTAAAAGC TT

1 **M**RGS**HHHHHH** GMASMTGGQQ MGRDLYDDDD KDPSSRSAAG TMAFKQEMGE NKKSPLIIAH
61 RGASGYLPEH TLEAKAYAYA LGADYLEQDI VLTKDNIPVI MHDPEIDTTT NVAQLFPNRA
121 RENGRYYATD FTLTELKSLN LSERFDPENK KPIYPNRFPL NEYNFKIPTL EEEIQFIQGL
181 NKSTGKNVGI YPEIKKPFWH KQQGKDISKI VIEILNKYGY KSKEDKIYLQ TFDFDELKRI
241 RKELGYQGKL IMLVGENDWN EAPTDYEYIK SEEGIAEVAK YSDGIGPWIP QIIIDGKITE
301 LTNLAHKYNI EVHPYTFRTD ALPSYVKNEN ELLDLLFNKA KVDGIFTDFT DTVMNFIKKE
361 F

pET24d_GlpQDIA

1 CC**ATG**GCA**CA TCACCATCAC CATCACCATC ACCATCAC**TT TAAGCAGGAA ATGGGCAGCA
61 ATAAAAAGAG CCCGCTGATT ATCGCGCACC GTGGCGCGAG CGGCTACCTG CCAGAACATA
121 CGCTGGAGGC AAAGGCATAC GCCTATGCGC TGGGCGCAGA CTACCTGGAA CAGGACATCG
181 TGCTGACCAA AGATAATATC CCGGTGATCA TGCACGATCC TGAAATTGAC ACGACCACTA
241 ACGTCGCCCA GCTCTTCCCG AACCGTGCTC GCGAAAACGG CCGTTACTAT GCTACTGATT
301 TCACCCTGAC TGAACTGAAA TCCCTCTCCC TGTCTGAACG TTTCGACCCA GAAAATAAAA
361 AGCCGATCTA CCCAAACCGC TTCCCGCTGA ACGAGTACAA TTTCAAAATC CCGACCCTGG
421 AGGAAGAGAT TAAATTCATC CAAGGCCTCA ACAAATCGAC CGGTCGCAAC GTAGGTATCT
481 ACCCGGAAAT TAAAAAGCCT TTTTGGCATA AACAGCAAGG CAAGGATATC AGCAAAATTG
541 TTATCGAAAT TCTGAACAAA TATGGTTATA AATCCAAAGA AGATAAAATC TACCTGCAGA
601 CTTTCGATTT CGATGAACTC AAGCGCATTC GTAAAGAACT GGGTTACCAG GGTAAACTTA
661 TCATGCTGGT AGGTGAAAAC GATTGGAATG AGGCTCCGAC GGACTATGAG TATATTAAAT
721 CCGAGGAAGG CATTGCAGAA GTGGCTAAAT ACTCTGACGG TATCGGCCCG TGGATCCCGC
781 AGATCATTAT CGACGGCAAA ATTACTGAAC TGACCTCTCT GGCCCATAAA TATAACATTG
841 AGGTACACCC GTACACCTTC CGTACCGACT CTCTGCCATC CTACGTTAAA AATGAGAACG
901 AACTTCTGGA CCTGCTGTTC AACAAAGCGA AAGTCGACGG CATCTTTACT GATTTCACTG
961 ACACCGTTAT GAACTTTATC AAAAAATGAT AAGAATTC

1 **M**A**HHHHHHHH HH**FKQEMGSN KKSPLIIAHR GASGYLPEHT LEAKAYAYAL GADYLEQDIV
61 LTKDNIPVIM HDPEIDTTTN VAQLFPNRAR ENGRYYATDF TLTELKSLSL SERFDPENKK
121 PIYPNRFPLN EYNFKIPTLE EEIKFIQGLN KSTGRNVGIY PEIKKPFWHK QQGKDISKIV
181 IEILNKYGYK SKEDKIYLQT FDFDELKRIR KELGYQGKLI MLVGENDWNE APTDYEYIKS
241 EEGIAEVAKY SDGIGPWIPQ IIIDGKITEL TSLAHKYNIE VHPYTFRTDS LPSYVKNENE
301 LLDLLFNKAK VDGIFTDFTD TVMNFIKK

Pairwise alignment of GlpQ protein sequences used in the Vienna and DIARECT laboratories

```
                  ....|....| ....|....| ....|....| ....|....| ....|....| ....|....|
                         5         15         25         35         45         55
pRSET-B_Bm_GlpQ   MRGSHHHHHH GMASMTGGQQ MGRDLYDDDD KDPSSRSAAG TMAFKQEMGE NKKSPLIIAH
pET24d_GlpQDia    MAHHHHHHHH ---------- ---------- ---------- -HH......S ..........


                  ....|....| ....|....| ....|....| ....|....| ....|....| ....|....|
                         65         75         85         95        105        115
pRSET-B_Bm_GlpQ   RGASGYLPEH TLEAKAYAYA LGADYLEQDI VLTKDNIPVI MHDPEIDTTT NVAQLFPNRA
pET24d_GlpQDia    .......... .......... .......... .......... .......... ..........

                  ....|....| ....|....| ....|....| ....|....| ....|....| ....|....|
                        125        135        145        155        165        175
pRSET-B_Bm_GlpQ   RENGRYYATD FTLTELKSLN LSERFDPENK KPIYPNRFPL NEYNFKIPTL EEEIQFIQGL
pET24d_GlpQDia    .......... .........S .......... .......... .......... ....K.....


                  ....|....| ....|....| ....|....| ....|....| ....|....| ....|....|
                        185        195        205        215        225        235
```

```
pRSET-B_Bm_GlpQ   NKSTGKNVGI YPEIKKPFWH KQQGKDISKI VIEILNKYGY KSKEDKIYLQ TFDFDELKRI
pET24d_GlpQDia    .....R.... .......... .......... .......... .......... ..........

                  ....|....| ....|....| ....|....| ....|....| ....|....| ....|....|
                    245        255        265        275        285        295
pRSET-B_Bm_GlpQ   RKELGYQGKL IMLVGENDWN EAPTDYEYIK SEEGIAEVAK YSDGIGPWIP QIIIDGKITE
pET24d_GlpQDia    .......... .......... .......... .......... .......... ..........

                  ....|....| ....|....| ....|....| ....|....| ....|....| ....|....|
                    305        315        325        335        345        355
pRSET-B_Bm_GlpQ   LTNLAHKYNI EVHPYTFRTD ALPSYVKNEN ELLDLLFNKA KVDGIFTDFT DTVMNFIKKE
pET24d_GlpQDia    ..S....... .......... S......... .......... .......... .........-

                  .

pRSET-B_Bm_GlpQ   F
pET24d_GlpQDia    -
```

Nucleotide- and amino acid sequences of Vsp1 of *B.* miyamotoi strain LB-2001. Expression vector pRSET-B was used for adding the His-tag at the N-terminus. Sites for restriction endonucleases are underscored. The starting codon (methionine) and the His-tag are marked by bold letters.

pRSET-B-Bm_Vsp1

```
  1 ATGCGGGGTT CTCATCATCA TCATCATCAT GGTATGGCTA GCATGACTGG TGGACAGCAA
 61 ATGGGTCGGG ATCTGTACGA CGATGACGAT AAGGATCCAT CATGTGGAAG TGGGGGACCG
121 GCACCTAAGG AAGGGCAGGC AGCAAAGGCT GATGGGACAG TCGTTGATTT AGTAAAAGTA
181 AGTAAAAAGA TAAAAGATGC GGTTGAGTTT GCAGCAAATG TAAAAGAAGT AGAGACTTTA
241 GTTAAGTCAA TAAATGAACT TGCTAAAGCT ATTGGAAAGA AAATTAAGTC CGATGGGCAG
301 TTTGATACTG AGTCAGGTAA AAATGGATCA TTGCTTGCAG GAGCACAAAG TATAATGTTA
361 GCTGTAAAAG CTAAGTTAGG ACAATTGGAT AATAAAGAAG GGATTTCTAC TGAACTAAAG
421 CAAAAGGTTA CTGATTCTAA GACAAAAACA GAAACTTTCT TAACTAAATT GAAAGACAAT
481 CACTCTGATC TTGGTAAAAA TGAAGCTACT GATGCTCATG CAAAAAGTGC TATAGATATA
541 ACTGATACTG GTACTAAAGA TAAAGGAACT TCTGAGCTTA TCGCTTTGAA TACATCAATT
601 AATGCTTTGT TAGAAACCGC TAATGATGAA GTAGAAGCCG CAATTAAGGC CCTTATAAAT
661 CCTTCTAAAG CATTAACTGC TGGTCAATCT TCATAATGTA AAGAGATAAA TAATTTGAGT
721 AATTGTTATA AGTTTAATTT TTAATTAAAA GGTAAGTAAC TGGGAAACTC GAG
```

```
  1 MRGSHHHHHH GMASMTGGQQ MGRDLYDDDD KDPSCGSGGP APKEGQAAKA DGTVVDLVKV
 61 SKKIKDAVEF AANVKEVETL VKSINELAKA IGKKIKSDGQ FDTESGKNGS LLAGAQSIML
121 AVKAKLGQLD NKEGISTELK QKVTDSKTKT ETFLTKLKDN HSDLGKNEAT DAHAKSAIDI
181 TDTGTKDKGT SELIALNTSI NALLETANDE VEAAIKALIN PSKALTAGQS S
```

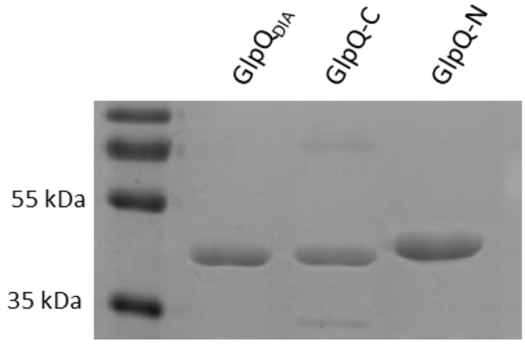

Figure S1. SDS-polyacrylamide gel electrophoresis of the different versions of GlpQ produced by us followed by staining with Coomassie Brilliant Blue. From left to right: GlpQDIA produced by DIARECT (38 kDa); GlpQ-C, GlpQ carrying a C-terminal 6xHis tag (~39 kDa); GlpQ-N, GlpQ carrying an N-terminal 6xHis tag (~41 kDa) both produced by the Vienna laboratory.

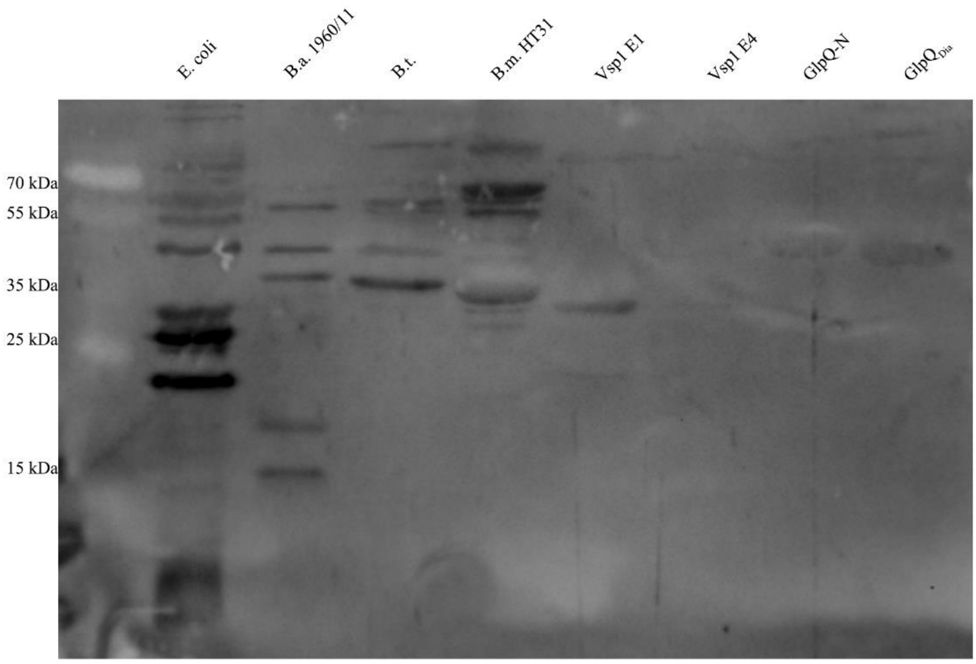

Figure S2. Immunoblot with the serum of the LNB patient, whose IgG shows weak reactivity against GlpQ. Bands visible for Vsp1 E1 and E4 are artefacts, probably due to impurities in the protein isolation (genuine bands for Vsp1 should be visible at ~24 kDa). *E. coli* strain Bl21(DE3)pLysS; B.a., *B. afzelii strain* 1960/11; B.t., *B. turicatae*; B.m.; HT31, *B. miyamotoi* strain HT31; Vsp1(E1 and E4), Vsp1 isolations with different purities; GlpQ-N, N-terminally 6xHis-tagged version of GlpQ produced in the Vienna laboratory; GlpQDIA, recombinant GlpQ produced by DIARECT.

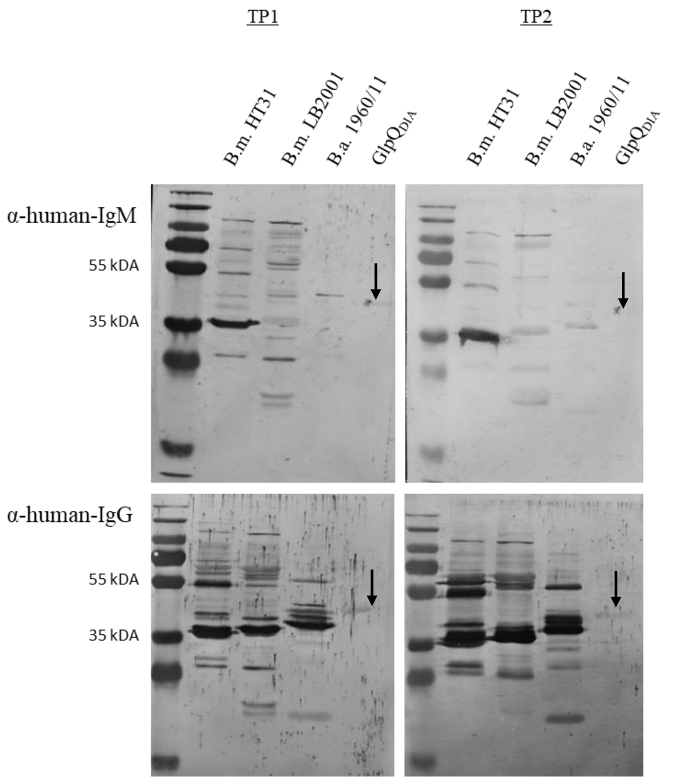

Figure S3. Serum samples of a *B. miyamotoi*-infected person from two different time points (TP). Infection was PCR confirmed at TP2. Sample collection at TP1 (13 weeks earlier) was not related to *B. miyamotoi* infection. The upper row was probed with α-human-IgM antibodies. The lower row was probed with α-human-IgG antibodies. B. m. HT31, *B. miyamotoi* strain HT31; B. m. LB2001, *B. miyamotoi* strain LB-2001; B.a. 1960/11, *B. afzelii* strain 1960/11; $GlpQ_{DIA}$, recombinant GlpQ produced by DIARECT. Arrows indicate the correct height of $GlpQ_{DIA}$
